# A Study Protocol for Validation and Implementation of Whole-Genome and -Transcriptome Sequencing as a Comprehensive Precision Diagnostic Test in Acute Leukemias

**DOI:** 10.3389/fmed.2022.842507

**Published:** 2022-03-24

**Authors:** Eva Berglund, Gisela Barbany, Christina Orsmark-Pietras, Linda Fogelstrand, Jonas Abrahamsson, Irina Golovleva, Helene Hallböök, Martin Höglund, Vladimir Lazarevic, Lars-Åke Levin, Jessica Nordlund, Ulrika Norèn-Nyström, Josefine Palle, Tharshini Thangavelu, Lars Palmqvist, Valtteri Wirta, Lucia Cavelier, Thoas Fioretos, Richard Rosenquist

**Affiliations:** ^1^Department of Immunology, Genetics and Pathology, Clinical Genomics Uppsala, Science for Life Laboratory, Uppsala University, Uppsala, Sweden; ^2^Department of Molecular Medicine and Surgery, Karolinska Institutet, Stockholm, Sweden; ^3^Clinical Genetics, Karolinska University Hospital, Solna, Sweden; ^4^Department of Clinical Genetics and Pathology, Office for Medical Services, Division of Laboratory Medicine, Lund, Sweden; ^5^Division of Clinical Genetics, Department of Laboratory Medicine, Lund University, Lund, Sweden; ^6^Clinical Genomics Lund, Science for Life Laboratory, Lund University, Lund, Sweden; ^7^Department of Clinical Chemistry, Sahlgrenska University Hospital, Gothenburg, Sweden; ^8^Department of Laboratory Medicine, Institute of Biomedicine, Clinical Genomics Gothenburg, Science for Life Laboratory, University of Gothenburg, Gothenburg, Sweden; ^9^Clinical Sciences, Queen Silvias Childrens Hospital, Gothenburg, Sweden; ^10^Department of Medical Biosciences, University of Umeå, Umeå, Sweden; ^11^Department of Medical Sciences, Uppsala University, Uppsala, Sweden; ^12^Department of Hematology, Oncology and Radiation Physics, Skåne University Hospital, Lund, Sweden; ^13^Department of Health, Medicine and Caring Sciences, Linköping University, Linköping, Sweden; ^14^Department of Medical Sciences and Science for Life Laboratory, Uppsala University, Uppsala, Sweden; ^15^Department of Clinical Sciences, Pediatrics, Umeå University, Umeå, Sweden; ^16^Women’s and Children’s Health, Uppsala University, Uppsala, Sweden; ^17^Department of Microbiology, Tumor and Cell Biology, Clinical Genomics Stockholm, Science for Life Laboratory, Karolinska Institutet, Solna, Sweden

**Keywords:** acute lymphoblastic leukemia, acute myeloid leukemia, whole-genome sequencing, whole-transcriptome sequencing, technical feasibility, diagnostic efficiency, clinical utility, health-economic evaluation

## Abstract

**Background:**

Whole-genome sequencing (WGS) and whole-transcriptome sequencing (WTS), with the ability to provide comprehensive genomic information, have become the focal point of research interest as novel techniques that can support precision diagnostics in routine clinical care of patients with various cancer types, including hematological malignancies. This national multi-center study, led by Genomic Medicine Sweden, aims to evaluate whether combined application of WGS and WTS (WGTS) is technically feasible and can be implemented as an efficient diagnostic tool in patients with acute lymphoblastic leukemia (ALL) and acute myeloid leukemia (AML). In addition to clinical impact assessment, a health-economic evaluation of such strategy will be performed.

**Methods and Analysis:**

The study comprises four phases (i.e., retrospective, prospective, real-time validation, and follow-up) including approximately 700 adult and pediatric Swedish AML and ALL patients. Results of WGS for tumor (90×) and normal/germline (30×) samples as well as WTS for tumors only will be compared to current standard of care diagnostics. Primary study endpoints are diagnostic efficiency and improved diagnostic yield. Secondary endpoints are technical and clinical feasibility for routine implementation, clinical utility, and health-economic impact.

**Discussion:**

Data from this national multi-center study will be used to evaluate clinical performance of the integrated WGTS diagnostic workflow compared with standard of care. The study will also elucidate clinical and health-economic impacts of a combined WGTS strategy when implemented in routine clinical care.

**Clinical Trial Registration:**

[https://doi.org/10.1186/ISRCTN66987142], identifier [ISRCTN66987142].

## Introduction

Genomic evaluation is emerging as an integral component of the diagnostic workup to facilitate diagnostic classification, risk stratification, and therapy selection in various cancers including hematological malignancies (1). To identify genetic variants that are associated with each type and subtype of hematologic cancer, such as single-nucleotide variants (SNVs), insertions and deletions (indels), oncogenic fusions, larger structural variations (SVs), and copy-number aberrations (CNAs), current standard of care (SoC) employs various, often laborious genetic techniques, including chromosome banding analysis, fluorescence *in situ* hybridization (FISH), genomic arrays, targeted next-generation sequencing (NGS), and reverse transcription polymerase chain reaction (RT-PCR). Although these methods provide clinically relevant information for individual patients, their detection/diagnostic capacities are hindered by low resolution, limited genomic coverage, and/or inability to identify novel genetic alterations, as well as the time and cost associated with performing multiple tests of the same sample. These challenges could potentially be overcome by using whole-genome sequencing (WGS), which covers the entire genome and thereby is capable of providing the most unbiased and comprehensive genetic information (2–5). Similarly, whole-transcriptome sequencing (WTS) that analyzes sequences of all expressed genes and has the ability to detect gene fusions (2, 6) is a method that may improve disease subclassification (6–9). Both WGS and WTS have so far been used primarily as research tools in hematological malignancies but are gaining interest as novel techniques to support diagnosis, prognosis, and therapy selection in a clinical setting (3–5).

Acute lymphoblastic (ALL) and acute myeloid leukemias (AML) are associated with clinically relevant SNVs, CNAs, and SVs including gene fusions (10). Since information on specific genetic aberrations today has a major impact on risk stratification and clinical management in both adult and pediatric acute leukemia, comprehensive genomic profiling by WGS and WTS is expected to be highly informative. Yet, studies that assess their clinical utility and health-economic impact compared to SoC diagnostics are limited.

The potential of a multi-modal sequencing approach as a clinical test was first evaluated in pediatric patients with various types of hematological malignancies and solid tumors at St. Jude Children’s Research Hospital (11). This study showed that the addition of WGS (30×) to combined whole-exome sequencing (100×)/WTS increased the sensitivity (from 78 to 98%) to identify pathogenic variants known from diagnostic testing (5). Using this diagnostic strategy, potentially actionable targets could also be identified that may facilitate future therapy selection and reduce treatment-related toxicities (12). Similarly, a study in myeloid cancers showed that, compared with conventional cytogenetics, tumor-only WGS (60×) analysis performed equivalently or better in identifying clinically relevant genetic variants and also changed risk stratification in a proportion (16%) of the patients (13). Together, these studies highlight the potential benefits of comprehensive precision diagnostic testing when used in routine practice, both to replace current SoC testing and to provide additional information beyond SoC. To advance clinical implementation of WGS and WTS, more studies that assess diagnostic efficiency/utility and health-economic impact of these methodologies are necessary.

## Aim

The aim of this national multi-center study performed by Genomic Medicine Sweden (GMS), in close collaboration with the Clinical Genomics Platform at Science for Life Laboratory (SciLifeLab), is to evaluate whether application of WGS and WTS combined, collectively termed WGTS, is technically feasible and can be implemented as an efficient clinical tool with a high diagnostic accuracy in patients with ALL and AML. Specifically, the combined workflow of WGTS will be compared with current SoC methodologies to identify potential benefits and challenges of WGTS implementation in a routine diagnostic setting. This study will also address whether WGTS will improve the diagnostic yield and patient management as well as if these assays combined are cost-effective. The ultimate goal of this study is to evaluate if WGTS can replace current diagnostic SoC methods of patients with ALL and AML across Sweden. This manuscript outlines the conceptual and experimental designs for the study.

## Methods and Analysis

### Study Design

**FIGURE 1:**
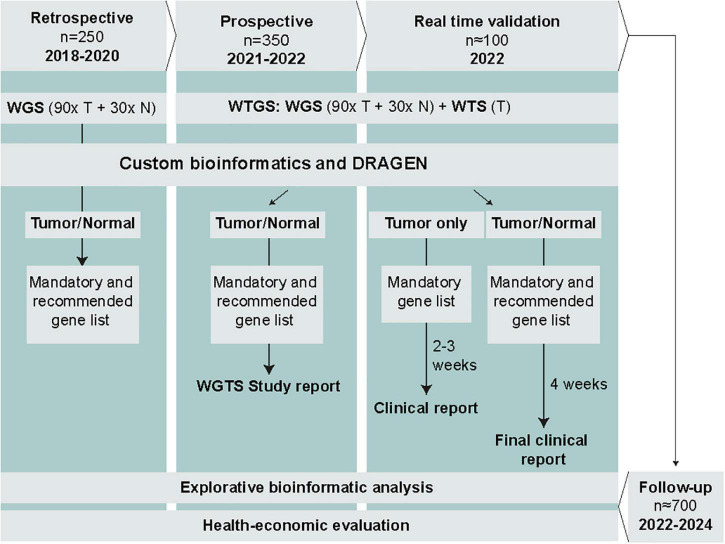
Overview of the study phases, planned data generation, analysis, and reporting. Results from SoC diagnostics will be collected in parallel for all patients. T, tumor; N, normal; WGS, whole-genome sequencing; WTS, whole-transcriptome sequencing.

The study is led by the Hematology Working Group within GMS. The study comprises 4 phases and includes a total of approximately 700 Swedish AML and ALL patients (Figure 1). The retrospective phase, involving WGS of tumor (90×) and normal (30×) samples from 150 adult AML patients and 100 pediatric ALL patients, has already been completed. Based on the retrospective data, we will assess technical feasibility and diagnostic efficiency of WGS to detect mandatory and highly recommended genetic variants in AML and ALL and determine to what extent WGS identifies additional clinically relevant genomic alterations not detected by SoC methods. The retrospective cohort was part of the SciLifeLab National Projects specifically focused on WGS, and therefore did not comprise WTS data. In the subsequent phases, we will complement with WTS to enable analysis of clinically relevant expressed fusion genes as well as explorative analysis of e.g., gene expression patterns and aberrant splicing (Supplementary Table 1).

The prospective phase encompasses WGTS of prospectively collected samples from approximately 350 adult and pediatric ALL and AML patients (Figure 1). WGS will be performed for tumor (90×) and normal (30×) samples, and WTS (50 million read pairs) for tumor samples only. The choice of sequence depth is based on recent recommendations and data from a previous study including acute leukemia samples (4, 14, 15). Samples will be processed in parallel with SoC methods but without a strict time limit for analysis and interpretation. The duration of this phase will be approximately 12 months, starting in May 2021.

The real-time validation phase comprises WGTS of prospectively collected samples from approximately 100 ALL and AML patients, as described for the prospective phase (Figure 1). The aim is to perform WGTS and report the results within the required time frame for genetic diagnostics of acute leukemias in parallel with SoC methods. The duration of this phase will be 3–6 months.

During the follow-up phase, patient outcome data will be recorded for those included in the prospective and real-time validation phases. At least 2 years of follow-up after the last included patient will be required for meaningful health-economic analysis.

### Endpoints

Primary and secondary endpoints are listed in Table 1.

**TABLE 1:** Primary and secondary endpoints.

Primary study endpoint (key phases)	Primary outcome measure
Diagnostic efficiency (retrospective and prospective phases)	Percentage of acute leukemia patients for whom all mandatory and highly recommended genetic variants found by SoC methods are also detected by WGTS*
Improved diagnostic yield (retrospective and prospective phases)	Percentage of acute leukemia patients for whom genetic variants relevant for classification or risk stratification are identified by WGTS* but not by SoC
**Secondary study endpoints (key phases)**	**Secondary outcome measure**
Technical feasibility (retrospective and prospective phases)	Percentage of patients for whom WGTS* analysis and interpretation is successful
Clinical feasibility for routine implementation (real-time validation phase)	Percentage of patients for whom WGTS analysis and interpretation is successful within a given time frame
Clinical utility (prospective and real-time validation phases)	Percentage of acute leukemia patients for whom patient management and/or therapy decision is/could be changed based on variants detected only by WGTS
Health-economic efficiency (all phases)	Micro-costing and cost effectiveness analysis of WGTS* compared to SoC

*For the retrospective phase, only WGS data are available.

#### Primary Endpoints

##### Diagnostic Efficiency

Diagnostic efficiency is based on

(1) the percentage of patients for whom WGS detects (or excludes) all mandatory and highly recommended variants identified by cytogenetics, FISH, genomic arrays, NGS panels and/or multiplex ligation-dependent probe amplification (retrospective and prospective phases);
(2) the percentage of patients for whom WTS detects all mandatory and highly recommended variants identified by RT-PCR or FISH (prospective phase); and
(3) the percentage of patients for whom combined WGTS detects all mandatory and highly recommended variants identified by SoC diagnostics (prospective phase).

In the real-time validation phase, percentages for WGS and WGTS will be recorded separately for tumor-only analysis (after 2–3 weeks) and tumor-normal analysis (after 4 weeks or when a germline sample has been analyzed).

##### Improved Diagnostic Yield

Improved diagnostic yield is determined by the percentage of acute leukemia patients for whom genetic variants relevant for classification or risk stratification are identified by WGTS but not by SoC methods (prospective phase). This includes inconclusive cases by SoC and genetic variants that were missed by SoC technologies but also those that were not actively screened for with SoC. In the real-time validation phase, improved diagnostic yield will be recorded separately for tumor-only and tumor-normal analysis.

#### Secondary Endpoints

##### Technical and Clinical Feasibility

Technical feasibility is assessed by the percentage of patients for whom WGTS analysis and interpretation are successfully completed without a strict time limit (prospective phase). Here we will also measure the percentage of samples where SoC tests or WGTS analysis were uninformative for mandatory and highly recommended variants.

Clinical feasibility is tested during the real-time validation phase and refers to the percentage of patients for whom WGTS analysis is successfully reported within the required time frame for genetic diagnosis of acute leukemias (i.e., 2 weeks for pediatric acute leukemias and 3 weeks for adult acute leukemias). Of note, this does not include targeted molecular tests that need to be performed in AML within the first days after diagnosis to decide on targeted therapy (e.g., *PML:RARA*, *FLT3*-ITD).

##### Clinical Utility

Clinical utility is measured by the percentage of patients for whom patient management or therapy decisions (according to national care programs and/or clinical trial protocols) are/potentially could be changed based on variants detected by WGTS, but not SoC diagnostics. Specifically, the percentage of patients for whom WGTS findings lead to targeted treatment options and/or a change in treatment options not identified by SoC will be recorded. This information will be retrieved from registries and retrospective analysis of medical records (prospective and real-time validation phases).

##### Health-Economic Evaluation

The health-economic evaluation consists of two analytic parts:

(1) A micro-costing analysis which is performed to estimate and compare the average cost of SoC and WGTS.
(2) Analysis of cost-effectiveness of WGTS (defined further below) in comparison with SoC will be estimated using economic simulation modeling.

### Participating Sites

The seven Swedish sites (Gothenburg, Linköping, Lund, Stockholm, Umeå, Uppsala, and Örebro) belonging to the Clinical Genomics Platform at SciLifeLab and corresponding Genomic Medicine Centers (GMCs) will participate in the study. While patients with acute leukemia will be recruited at all sites, sample processing, sequencing, and data analysis will be performed centrally at select locations (Supplementary Table 2).

### Study Population

#### Population and Sample Size

The retrospective cohort includes samples from 150 AML patients and 100 ALL patients for whom WGS has already been performed. For the prospective and real-time validation phases, the aim is to include a total of 450 consecutive adult and pediatric patients with a confirmed diagnosis of ALL or AML from any of the participating sites. This number corresponds approximately to the number of patients diagnosed with acute leukemias on average in Sweden within one year. The study will end after 15-18 months (depending on the patient recruitment rate) when the sample size has reached 450. The estimate of sample size at each site, based on the average number of patients diagnosed per year, is shown in Supplementary Table 3.

#### Inclusion Criteria (the Prospective and the Real-Time Validation Phases)

All patients diagnosed with acute leukemia and analyzed with genetic SoC methods will be offered inclusion. Patients will be included if the patient/guardians provide informed written consent and as long as sufficient tumor material is available for SoC tests and WGS.

### Sample Preparation

A bone marrow sample will be used for chromosome banding and FISH analyses. From the same sample, DNA and RNA will be extracted using standard procedures at each participating site. In the event of limited material from a bone marrow sample, peripheral blood may be used. The expected tumor purity in bone marrow as well as peripheral blood samples is >80–90%. WGTS of leukemic samples will be performed using surplus DNA and RNA that remain after SoC tests in most cases, without requiring additional sampling.

As germline material, a skin biopsy will be collected from which fibroblasts are cultured and DNA prepared. Alternatively, T-cells are isolated (adult AML) or a remission sample (taken at day 29) is used (adult ALL) as an alternative source of germline DNA. If a patient does not achieve remission, germline DNA will be extracted from a buccal swab sample also collected at day 29.

### SoC Genetic Diagnosis

The SoC methods used in AML include chromosome banding analysis to identify numerical and structural chromosomal aberrations, targeted FISH analysis to investigate stratifying structural variants, targeted NGS panel [e.g., GMS Myeloid custom panel (Twist Bioscience, South San Francisco, CA, United States) or Archer VariantPlex Myeloid panel (ArcherDx, Boulder, CO, United States)] and fragment analysis for selected mutations. Some of the fusion genes detected by FISH can alternatively be detected by RT-PCR. Supplementary Table 1 shows which types of genetic alterations are typically detected with which method.

The SoC methods used in ALL includes chromosome banding analysis, FISH to identify fusion genes and/or genomic arrays to identify CNAs. For pediatric patients and young adults with ALL, multiplex ligation-dependent probe amplification may also be performed to detect CNAs.

The methods included in SoC at each site are listed in Supplementary Tables 5–9. Since SoC is determined by national care programs and/or clinical study protocols, each site will analyze the same set of genetic alterations. As an example, mandatory genetic variants that must be detected or excluded in adult AML, according to current treatment protocols/guidelines, as well as highly recommended recurrent genetic variants are listed in Supplementary Table 4 (16).

### WGTS Protocol

All samples, regardless of tumor content, will be analyzed. WGS libraries will be prepared with the TruSeq PCR-Free kit (Illumina, San Diego, CA, United States). WTS libraries will be prepared with the TruSeq Stranded mRNA kit or Illumina Stranded mRNA Prep (Illumina). Input requirements are 1200 ng DNA at a concentration of 25 ng/μl and 500 ng RNA at a concentration of 10 ng/μl (RIN value ≥ 7). If sufficient material is unavailable, individual sequencing centers may use alternative PCR-free methods with lower input requirements. Sequencing will be performed using the NovaSeq platform (Illumina) with paired-end 150 bp read length. For WGS, leukemia samples will be sequenced to a target of 90× coverage and the matched germline samples to 30× coverage. For WTS, we aim to generate 50 million read-pairs or more for each leukemia sample.

### Bioinformatic Analysis

Bioinformatic analysis of WGTS data will be performed at each sequencing site. The bioinformatic pipelines at each site are outlined in Supplementary Table 10. Further development and optimization will be performed continuously throughout the study. Code will be made available in public repositories (Supplementary Table 10). GRCh38 will be used as the reference genome at all sites. In parallel, all sequencing data will be analyzed with the Illumina best practice workflow DRAGEN. This analysis will be performed in Stockholm and Lund; raw sequencing data generated at other sites will be transferred to either Stockholm or Lund for analysis (Supplementary Table 2).

### Data Interpretation

In all phases, data analysis and interpretation will be performed in two steps:

1. Targeted analysis of genes/variants that are mandatory to include or exclude in SoC. The lists of mandatory genetic variants for AML (Supplementary Table 4) and ALL were prepared based on the WHO classification (1) and current clinical study protocols and national/European guidelines.
2. Targeted analysis of an extended list of highly recommended genes/variants that is curated 1–2 times per year by the GMS Hematology Working Group. This list includes additional variants that are determined to be clinically relevant based on literature and other classifications (Supplementary Table 4).

In the retrospective phase, matched tumor-normal WGS data will be analyzed to identify mandatory and highly recommended genetic variants in AML (Supplementary Table 4) and ALL, while the prospective phase also includes tumor WTS data.

In the real-time validation phase, tumor-only analysis and interpretation will be performed according to step 1 within 2 weeks for pediatric acute leukemias and 3 weeks for adult acute leukemias, followed by tumor-normal analysis and final interpretation according to step 2 within 4 weeks (except for adult ALL where the remission sample will be collected at day 29).

After the completion of patient recruitment, a retrospective explorative research analysis of the entire WGS and WTS datasets will be performed to identify novel genomic aberrations of clinical interest.

### Reporting

For the whole study duration SoC will be the primary data source for subtype classification and the study data will be regarded as complementary information. Genetic variants of diagnostic, prognostic, and/or predictive impact detected by WGTS will be interpreted and recorded according to the standard procedure at each participating center.

In the real-time validation phase, all mandatory variants detected by WGTS will be included in the first clinical report [after 2 (ALL) or 3 (AML) weeks], whereas highly recommended variants will be included the final clinical report (after 4 weeks), and may potentially influence classification/risk stratification and treatment.

In the prospective phase, clinically relevant variants that are detected by WGTS, but not by SoC, will be reported to the hematologist/pediatric oncologist as a complementary clinical report after being verified with another clinically validated method (e.g., FISH, PCR or NGS-based gene panels). Thus, also in this phase, such variants may influence risk classification and treatment.

### WGTS Study Report

During the prospective and real time validation phases, we will prepare a WGTS study report for each patient with the following information:

• Availability of materials for SoC and WGTS.
• Sequencing metrics (sequence depth, sequence coverage, and other QC data).
• Mandatory and highly recommended variants identified by both SoC and WGTS.
• Mandatory and highly recommended variants that are identified by WGTS but not SoC, and if available results of verification experiments.
• Mandatory and highly recommended variants that are identified by SoC but not WGTS, and if available results of verification experiments.
• Potential impact of WGTS findings on classification, risk stratification and therapy selection.
• For the real-time validation phase, turnaround time in calendar days from sample collection to first clinical report (based on tumor-only analysis) and to the final report (tumor/normal analysis).

During the real-time validation phase, in addition to a WGTS study report, we will include WGTS findings in the clinical report according to the standard clinical reporting practice. Also, national review meetings, including physicians, clinical scientists, and bioinformaticians will be held on a regular basis to discuss the findings and their clinical interpretation (in particular, if WGTS detects clinically relevant genomic variants not identified by SoC or if WGTS and SoC show conflicting results) and to harmonize the reports between sites.

We have also prepared standardized templates for collection of metadata (e.g., sample data, sequencing protocol, sequencing platform, genome version, software and parameters used for variant calling, annotation and interpretation, etc.) to ensure that all sites collect the same information.

### Health-Economics Analysis

The micro-costing analysis will be performed initially at two sites and ultimately at all four sites where WGTS is carried out (Gothenburg, Lund, Stockholm, and Uppsala). A generic micro-costing spreadsheet will be developed and applied to Swedish genetic laboratories estimating the average cost of SoC and WGTS. The micro-costing will capture the economic burden to each laboratory; including the costs of personnel, equipment/material, bioinformatic analysis and interpretation, storage and compute costs, test consumables as well as platforms used (for WGTS) and potentially iterative testing. An activity-based costing model will be applied to calculate the total cost of the included diagnostic techniques. The analysis will take into account that some rapid conventional testing will be required in parallel to WGTS.

Cost-effectiveness of WGTS in comparison with SoC will be estimated using economic simulation modeling where the value of WGTS in quality adjusted life years is driven by the proportion of patients for whom management or therapy decision is/could be changed based on variants detected by WGTS, but not by SoC diagnostics. The outcome of patients where WGTS led to changes in risk stratification will be compared to the outcome of patients in risk groups defined by SoC only.

Based on literature, study outcomes (i.e., improved diagnostic yield and clinical utility), and registry data (e.g., the Swedish Blood Cancer Register, the National Patient Register, and the Swedish Prescribed Drug Register from the National Board of Health and Welfare), a simulation model will be built to estimate the diagnostic and the long-term cost for AML and ALL patients. The model will also be able to estimate the long-term health outcomes and the incremental cost effectiveness ratio of WGTS vs. SoC.

### Data Storage

Sequencing data will initially be stored at each university hospital/university and later on at the National Genomic Platform that is under development within GMS. Sequencing data from pediatric patients will also be stored at the Childhood Tumor Bank. In conjunction with publication, data will be stored at the European Genome-phenome Archive with restricted access. A data access committee that evaluates data requests will be formed within GMS.

### Statistical Analysis

In this study, our goal is to investigate all the patients with adult and pediatric acute leukemias who are diagnosed in Sweden during 12 months, i.e., 450 patients. Because a small proportion of patients (estimated to be less than 10%) is expected to be ineligible for the study (e.g., due to the unavailability of samples required for WGTS or the lack of consent to the study), we will continue recruiting for up to 18 months to ensure the inclusion of 450 patients in the study. Bivariate relationships between variables will be assessed using χ^2^ and unpaired *t*-tests. Diagnostic efficiency, sensitivity and specificity of WGTS for detection of mandatory and highly recommended genetic variants, as compared to SoC methods, will be assessed.

## Ethics and Dissemination

The application of WGS may potentially generate incidental or secondary findings of genetic variants linked to inherited disease. Notably, however, this study will not actively search for germline variants not related to hematologic malignancies. In the consent form, adult patients have the opportunity to opt in or out to receive information regarding unsolicited findings on inherited diseases beyond hematological conditions.

The study protocol was approved by the Swedish Ethical Review Authority for adult (2020-06673) and pediatric (2021-04135) acute leukemia patients. Data processing as well as the dissemination and exploitation of the study findings will take place in full compliance with EU data protection law.

## Discussion

This GMS multi-center study, performed in close collaboration with the Clinical Genomics platform at SciLifeLab, will investigate diagnostic efficiency, improved diagnostic yield, technical and clinical feasibility, and clinical utility of a combined WGTS workflow, as a routine clinical test for patients with acute leukemias and determine whether WGTS may replace current, often laborious SoC technologies. Specifically, the study aims to determine whether all mandatory genetic aberrations stipulated in the treatment protocols/national guidelines can be detected by WGTS within a given time frame. The only exceptions are targeted molecular tests that need to be performed within days after diagnosis. Additionally, a health-economic evaluation will be conducted including both a micro-costing and a cost-effectiveness analysis.

Clinical grade validation of short-read WGS in chronic lymphocytic leukemia (as a part of the Chronic Lymphocytic Leukemia Genomics England Pilot) demonstrated a high concordance (96%; 79/82 variants) between WGS and targeted NGS for SNV and indel detection (17). Concordance in CNA detection was slightly lower between WGS and FISH (87%; 26/30) or genome-wide high-resolution arrays (93%; 52/56) (17). Many of the discrepancies related to genetic alterations not covered by targeted sequencing or germline mutations filtered out and not reported by WGS (17). A recent study involving a large cohort of patients diagnosed with myeloid malignancies also showed that WGTS accurately detected genetic variants of clinical relevance and provided correct subtype classification (18). Furthermore, a head-to-head comparison of WGS (tumor-only, 60×) analysis to conventional cytogenetics and targeted sequencing not only confirmed accurate genomic profiling by WGS but also showed that WGS identified additional clinically relevant events in 17% (40/235) of patients and reclassified risk group assignments in 16% (19/117) of patients (13). Shallow WGS and WTS also demonstrated greater sensitivity in CNA detection and fusion detection in AML patients, respectively, compared with conventional cytogenetic analysis (19); concordance was high between cytogenetic analysis and shallow WGS (approximately 96%) or WTS (approximately 99%). Likewise, clinical value of WGS/WGTS in routine care of patients with solid tumors has been shown or is being investigated; e.g., the Hartwig Medical Foundation (20), the MASTER (21), and the WIDE (22) studies. Together, these findings substantiate the ability of WGTS to successfully support comprehensive genomic profiling for precision diagnostics.

Currently, there is a debate regarding the appropriate sequencing depth as well as whether normal sample should be included in the WGS analysis. Yet, current consensus for correct somatic variant calling by WGS are read depths of 90–100× for tumor samples and 30× for the normal control (15). For clear and reliable distinction between germline and somatic variants, comparative analysis of matched tumor and normal samples is also considered imperative (15). Based on the improved diagnostic yield reported by the study of Duncavage et al. that performed tumor-only analysis with WGS at 60× target coverage, our tumor-normal analysis using WGS at 90× coverage is expected to demonstrate a higher diagnostic performance. Our findings will also help address the current lack of standardized sequencing parameters and metrics for WGS. Furthermore, with the notable advantages of WTS such as oncogenic gene fusion detection and improved disease subclassification (6), additional diagnostic value gained by integration of WTS to WGS will be assessed in our study.

In the study by Duncavage et al., the cost of a WGS-based diagnostic approach was estimated to be $1,300–$1,900 (13). However, since the authors of this study performed lower depth of sequencing (60×) than what is currently recommended (15), the actual price could be higher and prohibitive for a realistic application of such sequencing methods in routine practice. They also used their in-house bioinformatics tools which does not take into account instrumentation and licensing fees by commercial solutions. These factors together likely caused underestimation of the total cost. Indeed, in a separate study, the cost of tumor-normal WGS (90× tumor coverage and 30× reference coverage) was determined to be >$3,000 (23). Additionally, the cost of WGS-based diagnostics may be higher at smaller clinical centers compared with larger institutions; in the former clinical setting where fewer patients are received, batching many patient samples to save cost is undesirable in the interest of reasonable/expected turn-around-time. Following the recommended sequencing strategy and using the matched tumor-normal samples (15), we aim to provide a more realistic estimation of the WGS/WTS-based diagnostic test.

To minimize the effects of inter-site heterogeneity in WGTS analysis, all sites will use the same type of sequencing instrument (NovaSeq) and reagents and the same gene lists will be used for variant prioritization. Although all sites will follow the same principles for data analysis, heterogenous in-house developed bioinformatic pipelines could potentially cause variability in variant calling and consequently affect study outcomes. Also, differences in variant filtering and interpretation procedures may give rise to differences. To evaluate this potential limitation, we will perform additional centralized data processing and analysis on all study samples, including analysis using the Illumina DRAGEN platform. This will allow us to pinpoint any major differences between sites due to the custom bioinformatic workflows. It will also provide an explorative option to identify any missed genetic aberrations in either the custom-developed tools or in DRAGEN, to guide further development of the analytic tools.

Our study is limited in that slightly different SoC methods will be used at the participating sites. Most of the SoC methods are very similar between the sites (e.g., chromosome analysis and FISH), whereas others may differ (e.g., gene panels). To ensure that SoC complies with the quality requirements all sites participate regularly in quality control rounds issued by CQAS and are yearly reviewed by the Swedish accreditation agency (SWEDAC). A significant fraction of the SoC investigations is also centrally reviewed annually to harmonize procedures. Thus, the final list of reported variants with SoC will be very similar regardless of which site performed the analysis and despite minor differences in methodology between sites. Moreover, annual updates of the national care programs will typically result in minor changes which are unlikely to have an impact on the current study. Similarly, the relevant clinical study protocols are not expected to change during the duration of the current study. Hence, although the SoC methods are slightly more heterogenous between sites than the WGTS approach (which is carried out at a smaller number of laboratories), the study will provide a direct comparison between the WGTS approach and the current SoC for each individual patient.

In summary, data from this national study will be used to (1) evaluate clinical performance of the integrated WGTS diagnostic strategy compared with SoC and (2) provide information regarding clinical and health-economic impacts of WGTS implementation in routine clinical care. We anticipate being able to disseminate our findings by 2022 and onward.

## Ethics Statement

The studies involving human participants were reviewed and approved by the Swedish Ethical Review Authority (2020-06673 and 2021-04135). Written informed consent to participate in this study was provided by the patient/guardians.

## Author Contributions

EB, GB, CO-P, LC, TF, and RR designed the study and wrote the manuscript. All authors contributed to the article and approved the submitted version.

## Conflict of Interest

The authors declare that the research was conducted in the absence of any commercial or financial relationships that could be construed as a potential conflict of interest.

## Publisher’s Note

All claims expressed in this article are solely those of the authors and do not necessarily represent those of their affiliated organizations, or those of the publisher, the editors and the reviewers. Any product that may be evaluated in this article, or claim that may be made by its manufacturer, is not guaranteed or endorsed by the publisher.
