# Supplementary material for: A Study Protocol for Validation and Implementation of Whole-Genome and -Transcriptome Sequencing as a Comprehensive Precision Diagnostic Test in Acute Leukemias
Source: Front Med (Lausanne). 2022 Mar 24;9:842507. doi: 10.3389/fmed.2022.842507 (PMC8987911; doi:10.3389/fmed.2022.842507)
Supplement: Supplementary file 1 [file Data_Sheet_1.docx]

Supplemental Material

**Supplemental Table 1.** Methods used for analysis of different types of genetic alterations.

| **Type of genetic variant** | **SoC methods** | **Analysed with WGS** | **Analysed with WTS** |
| --- | --- | --- | --- |
| SNVs/indels | NGS gene panel, fragment analysis | Yes | No^3^ |
| CNA | MLPA, genomic array | Yes | No |
| SV | CBA | Yes | No |
| Fusion gene | FISH, RT-PCR, targeted RNA-seq | Yes^2^ | Yes |
| Numerical chromosomal aberrations | CBA | Yes | No |
| Gene expression^1^ | - | No | Yes |
| Aberrant splicing^1^ | - | No | Yes |

^1^ Analysis of gene expression and aberrant splicing will be performed as part of the explorative study.

^2^ WGS can detect breakpoints of fusion genes but not determine whether or not a fusion gene is expressed.

^3^ WTS data will be used to confirm splice variants when needed.

**Supplemental Table 2:** Sample processing and data analysis in the study.

| **Patient recruitment** | **DNA/RNA extraction** | **Sequencing** | **DRAGEN analysis** | **Custom analysis** | **Clinical interpretation** |
| --- | --- | --- | --- | --- | --- |
| **Gothenburg** | on-site | WGS on-site, WTS in Lund | Lund | on-site | on-site |
| **Linköping** | on-site | MLL Munich Leukemia Laboratory | Lund | Lund | Lund |
| **Lund** | on-site | on-site | on-site | on-site | on-site |
| **Stockholm** | on-site | on-site | on-site | on-site | on-site |
| **Umeå** | on-site | Stockholm/ Uppsala | Stockholm | Stockholm/ Uppsala | Stockholm/ Uppsala |
| **Uppsala** | on-site | on-site | Stockholm | on-site | on-site |
| **Örebro** | Uppsala | Uppsala | Stockholm | Uppsala/ Örebro | Uppsala/ Örebro |

**Supplemental Table 3:** Expected sample size in the prospective and real-time validation phases at each site.

| **Site** | **No. of patients** |
| --- | --- |
| Uppsala (incl. Örebro) | 90 |
| Lund | 90 |
| Linköping | 45 |
| Stockholm | 90 |
| Umeå | 45 |
| Gothenburg | 90 |
